# Molecular cloning and expression analysis of *KIN10* and cold-acclimation related genes in wild banana ‘Huanxi’ (*Musa itinerans*)

**DOI:** 10.1186/s40064-015-1617-z

**Published:** 2015-12-30

**Authors:** Weihua Liu, Chunzhen Cheng, Gongti Lai, Yuling Lin, Zhongxiong Lai

**Affiliations:** Institute of Horticultural Biotechnology, Fujian Agriculture and Forestry University, Fuzhou, 350002 Fujian China

**Keywords:** Cold acclimation, Gene expression, *KIN10*, *Musa itinerans*, Sucrose

## Abstract

Banana cultivars may experience chilling or freezing injury in some of their cultivated regions, where wild banana can still grow very well. The clarification of the cold-resistant mechanism of wild banana is vital for cold-resistant banana breeding. In this study, the central stress integrator gene *KIN10* and some cold-acclimation related genes (*HOS1* and *ICE1s*) from the cold-resistant wild banana ‘Huanxi’ (*Musa itinerans*) were cloned and their expression patterns under different temperature treatments were analyzed. Thirteen full-length cDNA transcripts including 6 *KIN10s*, 1 *HOS1* and 6 *ICE1s* were successfully cloned. Quantitative real-time PCR (qRT-PCR) results showed that all these genes had the highest expression levels at the critical temperature of banana (13 °C). Under chilling temperature (4 °C), the expression level of *KIN10* reduced significantly but the expression of *HOS1* was still higher than that at the optimal temperature (28 °C, control). Both *KIN10* and *HOS1* showed the lowest expression levels at 0 °C, the expression level of *ICE1*, however, was higher than control. As sucrose plays role in plant cold-acclimation and in regulation of KIN10 and HOS1 bioactivities, the sucrose contents of wild banana under different temperatures were detected. Results showed that the sucrose content increased as temperature lowered. Our result suggested that KIN10 may participate in cold stress response via regulating sucrose biosynthesis, which is helpful in regulating cold acclimation pathway in wild banana.

**Electronic supplementary material:**

The online version of this article (doi:10.1186/s40064-015-1617-z) contains supplementary material, which is available to authorized users.

## Background

Banana (*Musa* spp.) is one of the most important nutrient-rich crops, staple foods and ornamental plants cultivated in tropical and subtropical regions where temperature is relatively high. Nonetheless, considerable interests still exit in exploring banana cold-resistant genes and developing cold tolerant banana cultivars due to the chilling or freezing injuries they might experience at some of their cultivated regions (Yang et al. 2012). Up to now, however, no effective method has yet been developed to effectively solve the cold injury problem.

Wild banana germplasm resources are abundant in China, where various studies have been conducted over the past 20 years (Liu et al. 2007, 2012; Lai et al. 2007). Wild banana species are more cold resistant than cultivated ones and can grow under relatively lower temperatures (Lai et al. 2007). The discovery of beneficial wild banana gene resources is consequently of great usefulness for cold-resistance breeding of cultivated banana.

Cold acclimation can dramatically increase freezing tolerance of plants and is very important for extending their adaptation areas (Zhang et al. 2009). It was reported that sucrose can enhance cold hardening of plants by regulating expression of cold-acclimation-associated genes such as *CBF* (*C*-*repeat/DRE*-*binding factor*), *ICE1* (*inducer of CBF expression1*), *HOS1* (*high expression of osmotically responsive gene 1*) and so on (Palonen and Junttila 2002; Rekarte-cowie et al. 2008). The expression of *CBF1*, was identified to be induced under cold stress (Lee et al. 2001). And its CBF AP2 motif combines with the CRT/DRE element in the COR promoter to enhance COR expression and ultimately plant cold tolerance (Chen et al. 2014). In *Arabidopsis*, three CBF genes, *CBF1*, *CBF2*, and *CBF3*, were found (Stockinger et al. 1997; Gilmour et al. 1998; Medina et al. 1999) and their functions in cold acclimation were also identified (Novillo et al. 2007). ICE1 is a transcription factor that can activate the expression of CBFs, thus function in regulating the cold-induced transcriptome and freezing tolerance (Chinnusamy et al. 2003). HOS1, a functional E3 ligase targeting ICE1 for ubiquitination-mediated ICE1 degradation, is the negative regulator of plant cold responses and its expression is regulated by osmotic potent changes (Dong et al. 2006). Sucrose can significantly influence the osmotic potential of plant cells (Palonen and Junttila 2002). Interestingly, the bioactivity of SNF1-related protein kinase catalytic subunit alpha 10/11 (KIN10/11), the central integrator of transcription networks in plant stress and energy signaling (Baena-González et al. 2007), can be inhibited by high concentrations of sucrose (Thalor et al. 2012). KIN10/KIN11, in reverse, can phosphate the key enzyme of sucrose biosynthesis (sucrose phosphate synthase) (Thalor et al. 2012). These correlations suggest that KIN10/KIN11 should function in cold hardening of plants. Thus far, however, studies of banana *KIN10*/*KIN11* and cold-acclimation related genes were rare due to the lack of sequence information.

Wild banana is widely distributed in all prefecture-level cities in Fujian Province, China (Lai et al. 2007). Among various germplasm resources, a wild banana population recently found in Huanxi, Fuzhou City, China, was found to be very tolerant to cold (Liu et al. 2012), making it very nice gene resources for cold-resistant genes and germplasm resources for cold-tolerant banana breeding. The release of Malaysian wild banana (*Musa acuminata*) genome data will be surely helpful for identification and characterization of these genes (D’Hont et al. 2012). So, in this study, *KIN10*, *HOS1* and *ICE1* (the target gene of *HOS1*) genes were identified by searching the Malaysian wild banana genome data and were cloned by RT-PCR and/or RACE technologies from cold-resistant wild banana ‘Huanxi’ (*Musa itinerans*). Their expression patterns under different temperature, together with the sucrose content in leaves, were also analyzed. Our study could be helpful in understanding the cold acclimation responses of wild banana under different temperature and in exploring the function of KIN10 in cold response.

## Results

In total, 6 *KIN10* (*KIN10*-*1*–*KIN10*-*6*), 1 *HOS1* and 6 *ICE1* (*ICE1*-*1*–*ICE1*-*6*) genes were successfully cloned from cold-resistant wild banana ‘Huanxi’. To the best of our known, this is the first report about cloning these genes from *Musa* spp. The generated sequences were submitted to GenBank, and the corresponding accession numbers were granted to KC127685, KC127686, KC127687, KC127688, KC127689, KC127690, JX678611, KC157569, KC157570, KC157571, KC157572, KC157573 and KC157574, respectively.

### Identification, characterization and bioinformatic analysis of *KIN10* genes from cold-resistant wild banana ‘Huanxi’

Multiple-sequence BLAST search revealed that *KIN10*-*1*, *KIN10*-*2* and *KIN10*-*3* had similar ORF sequences that were 95.51 % identical to the *KIN10* of Malaysian wild banana (*Musa acuminata*) (GSMUA_Achr10 G09220_001). But the ORF sequences of *KIN10*-*4*, *KIN10*-*5* and *KIN10*-*6* shared lower identity (only 76.58 %). These sequence variations may be due to differences between genes or species. Bioinformatics prediction result revealed that all the 6 *KIN10s* were basic, hydrophilic, and unstable proteins possessing transmembrane domains with predicted location in the nucleus or in membranes. Moreover, 21–26 phosphorylation sites were found in KIN10s (Table 1). Observed variations in the number and position of these phosphorylation sites suggest that some of their potential functions may be different. The KIN10s possessed 10–13 conserved domains, most of which were protein kinase domains (Additional file 1: Table S1). Phylogenetic analysis of KIN10 sequences generated the tree shown in Additional file 2: Figure S1. Besides the Malaysian wild banana KIN10, *Phoenix dactylifera* KIN10 and *Elaeis guineensis* KIN10 showed the closest relationship with wild banana ‘Huanxi’ KIN10s.

**Table 1:** Information of KIN10s, HOS1 and ICE1s proteins in wild banana ‘Huanxi’

Protein name	Amino acid no.	Protein property	Phosphorylation site no.	Predicted subcellular location
KIN10-1	491	Hydrophilic, basic	26	Nucleus
KIN10-2	513	Hydrophilic, basic	23	Plasma membrane
KIN10-3	513	Hydrophilic, basic	22	Plasma membrane
KIN10-4	506	Hydrophilic, basic	22	Plasma membrane
KIN10-5	513	Hydrophilic, basic	21	Plasma membrane
KIN10-6	513	Hydrophilic, basic	22	Plasma membrane
ICE1-1	547	Hydrophilic, acidic	23	Nucleus
ICE1-2	541	Hydrophilic, acidic	23	Nucleus
ICE1-3	542	Hydrophilic, basic	30	Nucleus
ICE1-4	536	Hydrophilic, basic	30	Nucleus
ICE1-5	503	Hydrophilic, acidic	20	Nucleus
ICE1-6	559	Hydrophilic, acidic	23	Nucleus
HOS1	967	Hydrophilic, acidic	57	Nucleus

### Identification, characterization and bioinformatic analysis of *HOS1* from cold-resistant wild banana ‘Huanxi’

The *HOS1* cDNA was 2926 bp long and contained a 2904 bp ORF encoding 967 amino acids. Multiple-sequence BLAST comparison showed *HOS1* from ‘Huanxi’ shared high similarity (93.95 %) with the Malaysian wild banana *HOS1* (GSMUA_Ach1G14640_001). The major difference between the two species was the presence of a 140 bp insertion in the upstream region of the ‘Huanxi’. On the basis of bioinformatics prediction analysis, HOS1 was shown to be a nuclear-localized, hydrophilic unstable protein without signal peptide. And 57 phosphorylation sites and a specific ELYS-like conserved domain were found in HOS1 (Table 1). Phylogenetic analysis of HOS1 sequences generated the tree shown in Additional file 3: Figure S3. Besides the Malaysian wild banana HOS1, *Phoenix dactylifera* HOS1 showed the closest relationship with wild banana ‘Huanxi’ HOS1.

### Identification, characterization and bioinformatic analysis of *ICE1* genes from cold-resistant wild banana ‘Huanxi’

Multiple-sequence BLAST search showed that the cloned *ICE1*-*1*–*ICE1*-*4* genes shared higher identity (97.52 %) with Malaysian wild banana *ICE1* (GSMUA_Achr10 G18380_001) compared with *ICE1*-*5* and *ICE1*-*6* (92.08 %). A 75 bp sequence, which was almost exactly the same size as that of *ICE1* introns in Malaysian wild banana, was missing from the middle region of ICE1-1–ICE1-4 in wild banana ‘Huanxi’. Other missing sequences in wild banana ‘Huanxi’ were a 16 bp sequence absent from the upstream region of *ICE1*-*3* and *ICE1*-*4* and a 19 bp sequence deleted from the termination codon region of *ICE1*-*1* and *ICE1*-*3*. *ICE1*-*5* and *ICE1*-*6* were 9 bp longer in wild banana ‘Huanxi’. Interestingly, compared with the Malaysian wild banana *ICE1*, *ICE1*-*5* of wild banana ‘Huanxi’ contained one more intron and one fewer exon and *ICE1*-*6* possessed two additional introns, which might be results of alternative splicing in evolution (Keren et al. 2010). According to bioinformatics prediction, the first four wild banana ‘Huanxi’ ICE1s encoded similar numbers of amino acid residues, whereas the number of amino acid residues encoded by ICE1-5 and ICE1-6 was quite different (Table 1). ICE1-1, ICE1-2, ICE1-5, and ICE1-6 were acidic proteins and ICE1-3 and ICE1-4 were basic. All six ICE1s were hydrophilic proteins with a specifically conserved basic-helix-loop-helix domain (IPR011598) as well as a non-integrated domain designated as PTH31945:SF0 and were predicted to be located in the nucleus. ICE5 and ICE6 featured an additional non-integrated conservative domain SSF55021 (Additional file 4: Table S2). The number of phosphorylation sites in ICE1s also varied from 20 to 30 among these 6 ICE1s (Table 1). ICE1s were predicted to be located in the nucleus and contained trans-membrane domains. Phylogenetic analysis of ICE1 sequences generated the tree shown in Additional file 5: Figure S2. Besides the Malaysian wild banana ICE1, the closest relationship was found between wild banana ‘Huanxi’ ICE1s and *Elaeis guineensis* ICE1.

### qRT-PCR analysis of *KIN10*, *HOS1* and *ICE1* genes in wild banana under low-temperature stress

**Fig. 1:**
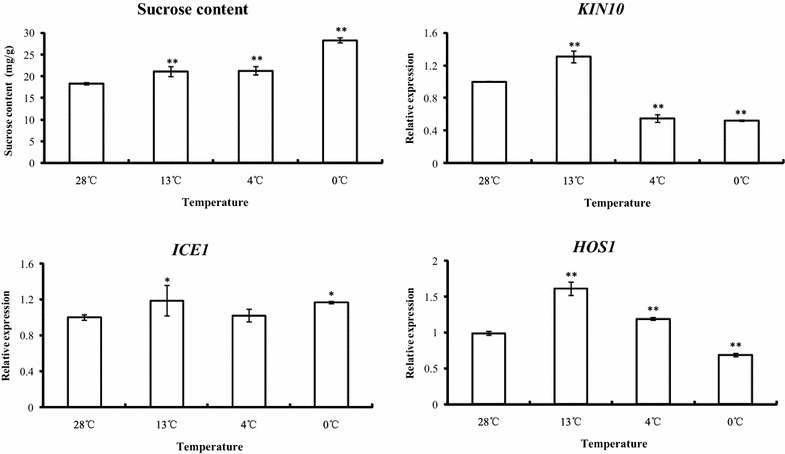
Changes of sucrose contents and expression levels of *KIN10*, *ICE1* and *HOS1* under different temperature treatments. Data was shown as mean ± SD of three independent experiments. *Asterisk* mark a statistical significant change with *p* value <0.05 (vs. control), *double asterisk* mark a very statistical significant change with *p* < 0.01 (vs. control)

As shown in Fig. 1, the expression levels of *KIN10*, *HOS1* and *ICE1* genes differed significantly under different temperatures. At the banana critical temperature of 13 °C, all these genes showed the highest expression levels. The expression levels of *KIN10* decreased significantly at 4 and 0 °C compared with control. Although the up-regulation level decreased, the expression level of *HOS1* at 4 °C was still higher than that of the control. At 0 °C, however, the *HOS1* expression level dropped to their lowest levels at 0 °C, a temperature at which *ICE1* expression levels were obviously increased.

### Sucrose contents in wild banana changed significantly under low-temperature stress

Sucrose contents of samples extracted from plants subjected to 0, 4, 13 and 28 °C for 36 h were shown in Fig. 1. Sucrose content rose markedly as the temperature dropped. Notably, the sucrose content in ‘Huanxi’ leaves at 4 °C was only about 1.16-fold higher than control. At 0 °C, however, the sucrose content was even 1.5-fold higher than that of the control.

## Discussion

Although banana cultivars grow in tropical and subtropical regions, they also suffer chilling or freezing damage at some part of its cultivated areas such as in Fujian, Guangdong, Guangxi and Yunnan provinces in China. Wild bananas are more tolerant to cold and thus are nice materials for exploring cold-resistant genes and clarifying the cold-acclimation mechanism.

Totally, 6 *KIN10*s, 1 *HOS1* and 6 *ICE1*s genes were successfully obtained. Sequence variations were found among *KIN10* and *ICE1* genes, which may be a consequence of evolutionary adaption to the environment: the Malaysian wild banana is distributed in tropical regions, whereas the wild banana ‘Huanxi’ is found at the northern margin of the southern subtropical region, and winter temperatures differ significantly between these two regions. Further research is needed to determine what, if any, functional differences are caused by the observed sequence differences.

Bioinformatic analysis also showed that all these genes are rich of phosphorylation sites, which suggest that phosphorylation may function a lot in plant cold resistance as phosphorylation was identified to play important role during the acquisition of freezing tolerance (Monroy et al. 1993; Komatsu et al. 1999; Schulze et al. 2012). Sequences analysis showed that protein-kinase domains of KIN10s were highly conserved, which might be the structural basis of the ‘master molecular switch’ in plant stress regulation (Baena-González et al. 2007). Observed variations in the number and position of these phosphorylation sites suggest that some of their potential functions may differ. The high number of conserved domains in wild banana KIN10s is unusual and reveals the diversity of these proteins’ unique functions. At the same time, many of these conserved KIN10 domains are protein kinase domains, the structural basis of the ‘master molecular switch’ in plant stress regulation.

Notably, the evolutionary alternative splicing events were identified in *ICE1* genes. As these genes are cold resistance-related functional genes from wild banana ‘Huanxi’, which is located in northern Fuzhou and often subject to low-temperature stress, we hypothesize that this alternative splicing phenomenon may be related to evolutionary adaptation to the environment to improve wild banana cold resistance. Whether any functional changes have occurred due to alternative splicing remains to be determined.

KIN10/KIN11 is an important regulator in various plant stress responses and its ability can be inhibited by high concentrations of sucrose (Baena-González et al. 2007; Thalor et al. 2012). The function of sucrose in plant cold acclimation (Palonen and Junttila 2002; Rekarte-cowie et al. 2008) indicated that KIN10/KIN11 might be also involved in plant cold acclimation. To prove this proposition, a new-found cold-resistant wild banana ‘Huanxi’ was used for cloning of the stress-related gene *KIN10* and cold-acclimation related genes, *HOS1* and *ICE1*.

To compare their expression under different temperatures and to elucidate their function in cold-acclimation, expression levels of these genes at the optimal temperature (28 °C), critical temperature (13 °C), chilling temperature (4 °C) and freezing temperature (0 °C) were detected. Banana is basically a tropical crop growing at temperatures ranging from 13 to 38 °C. Below 13 °C or above 38 °C, banana growth ceases and 13 °C is thought to be the critical low temperature for banana growth (Nelson et al. 2006). Our study revealed that the relative expressions of *KIN10s*, *HOS1* and *ICE1s* all reached the highest levels at the banana critical temperature. KIN10 could promote catabolic processes and suppress anabolic processes (Thalor et al. 2012), the up-regulation of *KIN10*, therefore, could be helpful in maintaining the energy balance and thus improve the cold resistance of wild banana at critical temperature. The expression of *KIN10* was significantly suppressed at both 4 and 0 °C. It was reported that once the cellular sucrose reached certain level, the activity of KIN10 would be suppressed (Baena-González et al. 2007; Thalor et al. 2012). At 4 °C, the sucrose content in ‘Huanxi’ leaves was about 21.2 mg/g fresh weight, which might be the threshold for sucrose content of wild banana ‘Huanxi’. KIN10 was also reported to be able to phosphorylate the key enzyme of sucrose biosynthesis, sucrose phosphate synthase (Thalor et al. 2012), so we inferred that KIN10 could participate in cold responses or cold-acclimation of wild banana through regulating sucrose biosynthesis.

The expression level of *HOS1*, an osmotically responsive gene, was still significantly higher than that of the control at 4 °C, which indicated that non-freezing low-temperature could induce the expression of HOS1. The sucrose content in leaves was higher at 4 °C than at 28 °C, suggesting that the up-regulation of HOS1 may be a result of increasing osmotic potential in plant cells. HOS1 is a negative regulator of low temperature signal transduction that mediates the ubiquitination and degradation of ICE1 (Dong et al. 2006), which is a regulator of freezing tolerance in plants (Chinnusamy et al. 2003). At freezing temperature (0 °C), expression of *HOS1* was suppressed and expression of *ICE1* was induced. The down-regulation of low temperature negative regulator and up-regulation of freezing tolerance regulator reflected well the increase of cold tolerance.

## Conclusion

**Fig. 2:**
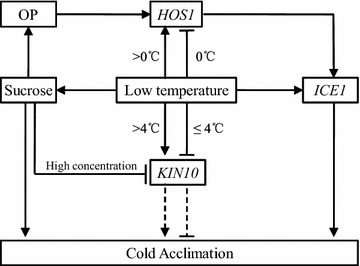
The involvement of *KIN10*, *HOS1*, *ICE1* and sucrose in the cold acclimation pathway of the cold-resistant wild banana ‘Huanxi’. *HOS1* high expression of osmotically responsive gene 1, *ICE1* inducer of CBF expression 1, *KIN10* SNF1-related protein kinase catalytic subunit alpha 10, *OP* osmotic potential

In conclusion, we successfully cloned several genes of KIN10, HOS1 and ICE1 from cold-resistant wild banana ‘Huanxi’. Expression analysis showed that expression of these genes and sucrose synthesis were significantly influenced by low temperature (Fig. 2): the expression of *ICE1* was induced by low temperature; the expression of *HOS1* was induced at non-freezing temperature but was suppressed at freezing temperature; the expression of *KIN10* was influenced by low temperature and it might participate in cold-response by regulating sucrose biosynthesis. Our study could provide clues for improving banana cold resistance and for cold-tolerant banana breeding.

## Methods

### Plant materials and treatments

Plants of cold-resistant wild banana ‘Huanxi’ (*Musa itinerans*) were obtained from the Wild Banana Germplasm Nursery of the Institute of Horticultural Biotechnology, Fujian Agriculture and Forestry University, Fuzhou, China. For temperature treatments, 25-day-old plantlets growing at 28 °C were subjected to 0, 4 and 13 °C for 36 h with plantlets growing at 28 °C as control.

### Gene cloning and sequence analysis

Homologous gene sequences in GenBank database (http://www.ncbi.nlm.nih.gov) and in the Malaysian wild banana (*Musa acuminata*) genome-wide data (http://banana-genome.cirad.fr) were downloaded and used as reference sequences for determination of the conserved regions and for primer design. Information of all the used primers was listed Additional file 6: Table S3. The gene cloning processes were shown in Additional file 7: Figure S4. The obtained cDNA sequences were all submitted to the GenBank database (http://www.ncbi.nlm.nih.gov).

Bioinformatics analysis of these obtained genes and their deduced protein sequences were performed according to Tian et al. (2015). Amino acid sequences of KIN10s, ICE1s, and HOS1 were deduced and analyzed with the ExPASy Protparam tool (Wilkins et al. 1999). BLAST searches were performed using the NCBI server. Multiple alignment and structural analysis of deduced proteins was performed using DNAMAN 6.0. Signal peptide and protein subcellular localization information were predicted using SignalP4.0 Server (Petersen et al. 2011) and PSORT (Nakai 2000) software, respectively. Protein transmembrane regions and orientations were predicted using the Tmpred program. Conserved domain fields were identified with InterProScan (http://www.ebi.ac.uk/interpro/scan.html) (Mitchell et al. 2015) and phosphorylation sites were predicted using the NetPhos 2.0 Server (http://www.cbs.dtu.dk/services/NetPhos/) (Wong et al. 2007). Phylogenetic analyses were performed using neighbor-joining in MEGA 5.02 (Tamura et al. 2011).

### qRT-PCR expression analysis of KIN10s, ICE1s and HOS1

To explore the relationship of these cold-resistance genes and the cold stress response mechanism in ‘Huanxi’ wild banana under low-temperature stress, we conducted a gene expression analysis using qRT-PCR. After low temperature treatment, leaves were sampled for total RNA extraction. qRT-PCR analyses were performed as described by Lin and Lai (2013) on Roche LightCycler480 (Roache, Basel, Switzerland) with *18S rRNA* as the internal control. Conserved regions of *KIN10s*, *HOS1* and *ICE1*-*1*–*ICE1*-*4* were used as templates for primer design. Three biological and technical replicates were made for each treatment. Primers used were listed in Additional file 8: Table S4.

### Sucrose content determination

The same extracts obtained from leaves exposed to 0, 4, 13 and 28 °C (control) for 36 h and used for the qPCR analysis were analyzed for sucrose content. The sucrose contents in leaves were measured by using the method of (Xue 1985).

## Additional files

10.1186/s40064-015-1617-z Conserved domains in KIN10s of wild banana ‘Huanxi’.
10.1186/s40064-015-1617-z Phylogenetic tree of the amino acid sequences of KIN10.
10.1186/s40064-015-1617-z Phylogenetic tree of the amino acid sequences of HOS1.
10.1186/s40064-015-1617-z Conserved domains in ICE1s of wild banana ‘Huanxi’.
10.1186/s40064-015-1617-z Phylogenetic tree of the amino acid sequences of ICE1.
10.1186/s40064-015-1617-z Information of primers used for cloning of *KIN10*s, *HOS1* and *ICE1*s from wild banana ‘Huanxi’.
10.1186/s40064-015-1617-z Cloning process of the cold-acclimation related genes in wild banana ‘Huanxi’.
10.1186/s40064-015-1617-z Information of primers used for qRT-PCR.

## Abbreviations

PCR: Polymerase chain reaction
KIN10s: SNF1-related protein kinase catalytic subunit alpha 10
HOS1: High expression of osmotic stress-regulated gene expression 1
CBF: CRT(C-repeat/dehydration response element)/DRE(dehydration responsive element)-binding factor
ICE1: Inducer of CBF expression 1
RT-PCR: Reverse transcription PCR
qRT-PCR: Quantitative real-time PCR
RACE: Rapid amplification of cDNA ends
DNA: Deoxyribonucleic acid
RNA: Ribonucleic acid
cDNA: Complementary DNA
bp: Base pair
OFR: Open reading frame
CDS: Coding sequence
UTR: Untranslated region
