# Supplementary material for: Molecular cloning and expression analysis of KIN10 and cold-acclimation related genes in wild banana ‘Huanxi’ (Musa itinerans)
Source: Springerplus. 2015 Dec 30;4:829. doi: 10.1186/s40064-015-1617-z (PMC4695468; doi:10.1186/s40064-015-1617-z)
Supplement: Supplementary file 2 — 10.1186/s40064-015-1617-z Phylogenetic tree of the amino acid sequences of KIN10. [file 40064_2015_1617_MOESM1_ESM.doc]

**Supplemental Table S1** Conserved domains in KIN10s of wild banana ‘Huanxi’

| Domain description* | Domain ID | KIN10-1 | KIN10-2 | KIN10-3 | KIN10-4 | KIN10-5 | KIN10-6 |
| --- | --- | --- | --- | --- | --- | --- | --- |
| Protein kinase, catalytic domain | PF00069 | + | + | + | + | + | + |
|  | PSS0011 | + | + | + | + | + | + |
| Serine/threonine-/dual-specificity protein kinase, catalytic domain | SM00220 | + | + | + | + | + | + |
| Serine/threonine-proteinkinase,activesite | PS00108 | + | + | + | + | + | + |
| Kinase-associated KIA | G3DSA:3.30.310.80 | _ | _ | _ | + | _ | _ |
|  | PF02149 | _ | + | + | + | + | + |
|  | PS50032 | _ | + | + | + | + | + |
|  | SSF103243 | _ | + | + | + | + | + |
| Protein kinase-like domain | SSF56112 | + | + | + | + | + | + |
| Ubiquitin-associated/translation elongation factor EF1B,N-terminal,eukaryote | PSS0030 | + | + | + | + | + | + |
|  | SM00165 | _ | + | + | + | + | + |
| Protein kinase, ATP Binding site | PS00107 | + | + | + | + | + | + |
| Tyrosine-protein kinase, catalytic domain | SM00219 | + | + | + | + | + | + |
| Unintegrated | G3DSA:1.10.510.10 | + | + | + | + | + | + |
|  | G3DSA:3.30.200.20 | + | + | + | + | + | + |
|  | PTHR24343 | + | + | + | + | + | + |
|  | PTHR24343:SF87 | + | + | + | + | + | + |
|  | G3DSA:1.10.8.10 | _ | _ | + | _ | _ | _ |

*: protein with the mentioned domain was marked ‘+’ , and protein without the mentioned domain was marked ‘-’.
