# Supplementary material for: Molecular cloning and expression analysis of KIN10 and cold-acclimation related genes in wild banana ‘Huanxi’ (Musa itinerans)
Source: Springerplus. 2015 Dec 30;4:829. doi: 10.1186/s40064-015-1617-z (PMC4695468; doi:10.1186/s40064-015-1617-z)
Supplement: Supplementary file 4 — 10.1186/s40064-015-1617-z Conserved domains in ICE1s of wild banana ‘Huanxi’. [file 40064_2015_1617_MOESM6_ESM.doc]

**Supplemental Table S3** Information of primers used for cloning of *KIN10*s, *HOS1* and *ICE1*s from wild banana ‘Huanxi’

| **Primer pairs name** | **Primer sequences**(5′-3′) | **Target** [**fragment**](javascript:void(0);) | **product sizes** (bp) |
| --- | --- | --- | --- |
| KIN10-1-conserved-F  KIN10-1-conserved-R | TGCATCCACATATCATCCATCTCTATG  TTATAGGACCCTAAGCTGTGCAAGG | The conserved sequence of *KIN10-1* | 1321 |
| KIN10-1-5-RACE-R1  KIN10-1-5-RACE-UPM | CTGCATAGTATCTGGTGGAGGT  TAATACGACTCACTATAGGGCAAGCAGTGGTATCAACGCAGAGT | 5’ sequence of *KIN10-1* | 710 |
| KIN10-1-5-RACE-R2  KIN10-1-5-RACE-5SNP | CATTACTCAAGCCGAAGTCAGC  AAGCAGTGGTATCAACGCAGAGT |
| KIN10-2-F  KIN10-2-R | GCGAGCGTATCCTCTCCTTATATG  TTATAGGACCCTAAGCTGTGCAAGG | Full-length  *KIN10-2* | 1676 |
| KIN10-3-F  KIN10-3-R | CGCTTCCTCGATTCTCTACAACGC  TTATAGGACCCTAAGCTGTGCAAGG | Full-length *KIN10-3* | 1600 |
| KIN10-4-conserved1-F  KIN10-4- conserved1-R | GGATCTAGCAAAGGAGGGGGTAGTG  GAGCATAAAGAATAACACCACAGC | The conserved sequence 1 of *KIN10-4* | 619 |
| KIN10-4-conserved2-F  KIN10-4- conserved2-R | GTTGCTATAAAAATCCTCAATCGTC  CATGTTATAGTGTCCGATCCTTTTCC | The conserved sequence 2 of *KIN10-4* | 1146 |
| KIN10-4-3-RACE-F1  KIN10-4-3-RACE-3P | GTTGCTATAAAAATCCTCAATCGTC  GCTGTCAACGATACGCTACGTAACG | 3’ sequence of *KIN10-4* | 932 |
| KIN10-4-3-RACE-F2  KIN10-4-3-RACE-3NP | ACATCCATGGTTCCAAATGCGTCTAC  CGCTACGTAACGGCATGACAGTG |
| KIN10-4-5-RACE-R1  KIN10-4-5-RACE-UPM | GAGCATAAAGAATAACACCACAGC  TAATACGACTCACTATAGGGCAAGCAGTGGTATCAACGCAGAGT | 5’ sequence of *KIN10-4* | 339 |
| KIN10-4-5-RACE-R2  KIN10-4-5-RACE-5SNP | CTTAACCTTGCCAAATGAGCCAATG  AAGCAGTGGTATCAACGCAGAGT |
| KIN10-5-F  KIN10-5-R | TCTCCTTATATGCCCATCCTCGGTG  TCATAGGACTCTAAGCTGCGCAAGGAAG | Full-length *KIN10-5* | 1664 |
| KIN10-6-F  KIN10-6-R | TCTCCTTATATGCCCATCCTCGGTG  TCATAGGACTCTAAGCTGCGCAAGGAAG | Full-length *KIN10-6* | 1663 |
| HOS1-conserved-F  HOS1-conserved-R | TAGGGACTTAAGCAGCTGTGGACGTTG  GCGACCCAGGCTTTTCAGAAGATGA | The conserved sequence of *HOS1* | 2713 |
| HOS1-3’RACE-F1  HOS1-3’RACE-UPM | TTAAATGTCTCTTTGCACCAGTCCTT  TAATACGACTCACTATAGGGCAAGCAGTGGTATCAACGCAGAGT | 3’ sequence of *HOS1* | 338 |
| HOS1-3’RACE-F2  HOS1-3’RACE-3NP | GTGAAGAATGCAGCAAGAGATGTGATG  AAGCAGTGGTATCAACGCAGAGT |
| HOS1-ORF-F  HOS1-ORF-R | GCTCTCGAAGGCGAGGAG  GCGACCCAGGCTTTTCAGAAGATGA | ORF of *HOS1* | 2926 |
| ICE1-1-ICE1-4-conserved1a-F  ICE1-1-ICE1-4-conserved1a-R | CAAAGGACTGTGTGAAACTCGATAG  GCTCTATCCATCTTGCTTATCTTGGG | The conserved sequence 1a of ICE1-1- ICE1-4 | 1510 |
| ICE1-1-ICE1-4-conserved1b-F  ICE1-1-ICE1-4-conserved1b-R | CAAAGGACTGTGTGAAACTCGATAG  GCTCTATCCATCTTGCTTATCTTGGG | The conserved 1b of I*CE1-1*~*ICE1-4* | 1511 |
| ICE1-1-ICE1-4-conserved2-F  ICE1-1-ICE1-4-conserved2-R | ATGGCGGAGAGGAGGAGGAGAAAGAAAC  AGAACACCCGGGTCTTTGCATTGCTC | The conserved sequence 2 of *ICE1-1*~*ICE1-4* | 509 |
| ICE1-1-ICE1-4-conserved3a-F  ICE1-1-ICE1-4-conserved3a-R | CCCTTAACTCCCACACTACCAACT  GGATAACTAATTCTAAGAGCTGCCG | The conserved sequence 3a of *ICE1-1*~ *ICE1-4* | 449 |
| ICE1-1-ICE1-4-conserved3b-F  ICE1-1-ICE1-4-conserved3b-R | CCCTTAACTCCCACACTACCAACT  GGATAACTAATTCTAAGAGCTGCCG | The conserved sequence 3b of *ICE1-1*~*ICE1-4* | 450 |
| ICE1-5-ICE1-6-conserved1-F  ICE1-5-ICE1-6-conserved1-R | AACCCCACACCGTCTTCTGTCTTCTT  CGGAGCTCAGATCGGGACAAC | The conserved sequence 1 of *ICE1-5* and *ICE1-6* | 630 |
| ICE1-5-ICE1-6-conserve2a-F  ICE1-5-ICE1-6-conserve2a-R | GACGACGACTGGTACCTGGG  GCAAAAGCTCTTTCAGGTACTCAATTG | The conserved sequence 2a of *ICE1-5* | 910 |
| ICE1-5-ICE1-6-conserve2b-F  ICE1-5-ICE1-6-conserve2b-R | GACGACGACTGGTACCTGGG  GCAAAAGCTCTTTCAGGTACTCAATTG | The conserved sequence 2b of *ICE1-6* | 1078 |
| ICE1-5-ICE1-6-conserve3-F  ICE1-5-ICE1-6-conserve3-R | GCTTTACATGCTTAGGTCTGTTGTTCC  TCATGACACTGTATTATCGAAGCC | The conserved sequence 3 of *ICE1-5* and *ICE1-6* | 529 |
