# Supplementary material for: Molecular cloning and expression analysis of KIN10 and cold-acclimation related genes in wild banana ‘Huanxi’ (Musa itinerans)
Source: Springerplus. 2015 Dec 30;4:829. doi: 10.1186/s40064-015-1617-z (PMC4695468; doi:10.1186/s40064-015-1617-z)
Supplement: Supplementary file 5 — 10.1186/s40064-015-1617-z Phylogenetic tree of the amino acid sequences of ICE1. [file 40064_2015_1617_MOESM2_ESM.doc]

**Supplemental Figure S1** Phylogenetic tree of the amino acid sequences of KIN10
