# Supplementary material for: Molecular cloning and expression analysis of KIN10 and cold-acclimation related genes in wild banana ‘Huanxi’ (Musa itinerans)
Source: Springerplus. 2015 Dec 30;4:829. doi: 10.1186/s40064-015-1617-z (PMC4695468; doi:10.1186/s40064-015-1617-z)
Supplement: Supplementary file 6 — 10.1186/s40064-015-1617-z Information of primers used for cloning of KIN10s, HOS1 and ICE1s from wild banana ‘Huanxi’. [file 40064_2015_1617_MOESM7_ESM.doc]

**Supplemental Figure S4** Cloning process of the cold-acclimation related genes in wild banana ‘Huanxi’. M: DL2000 marker, M’: DL5000 marker, 1 and 2: PCR result of the conserved sequence and the 5’ RACE sequence of *KIN10-1*; 3 and 4: PCR result of full-length *KIN10-3* and *KIN10-2*; 5 and 6: PCR result of the conserved sequence 1a and 2 of *KIN10-4*; 9 and 12: PCR result of the 3’RACE and 5’ RACE sequence of *KIN10-4*; 13 and 14: PCR results of full-length *KIN10-5* and *KIN10-6*; 15-17: PCR results of the conserved sequence, 5’RACE sequence and the ORF sequence product of *HOS1*; 18, 19, 21, 22 and 24: PCR results of the conserved 1a, 1b, 2, 3a and 3b sequence products of *ICE1-1*-*1-4*; 25: PCR result of the conserved sequence 1 of *ICE1-5* and *ICE1-6*; 28: PCR result of the conserved sequence 2a of *ICE1-5*; 29. PCR result of the conserved sequence 2b of *ICE1-6*; 31: PCR result of the conserved sequence 3 of *ICE1-5* and *ICE1-6*; 7, 8, 10, 11, 20, 23, 26, 27 and 30: nonspecific PCR products
