# Supplementary material for: Molecular cloning and expression analysis of KIN10 and cold-acclimation related genes in wild banana ‘Huanxi’ (Musa itinerans)
Source: Springerplus. 2015 Dec 30;4:829. doi: 10.1186/s40064-015-1617-z (PMC4695468; doi:10.1186/s40064-015-1617-z)
Supplement: Supplementary file 7 — 10.1186/s40064-015-1617-z Cloning process of the cold-acclimation related genes in wild banana ‘Huanxi’. [file 40064_2015_1617_MOESM4_ESM.doc]

**Supplemental Table S2** Conserved domains in ICE1s of wild banana ‘Huanxi’

| Domain description | Domain ID | ICE1-1 | ICE1-2 | ICE1-3 | ICE1-4 | ICE1-5 | ICE1-6 |
| --- | --- | --- | --- | --- | --- | --- | --- |
| Helix-loop-helix domain | G3DSA:4.10.280.10 | + | + | + | + | + | + |
|  | PF00010 | + | + | + | + | + | + |
|  | SM00353 | + | + | + | + | + | + |
|  | PS50888 | + | + | + | + | + | + |
|  | SSF47459 | + | + | + | + | + | + |
| unintegrated | PTHR31945 | + | + | + | + | + | + |
|  | PTHR31945:SF0 | + | + | + | + | + | + |
|  | SSF55021 | _ | _ | _ | _ | + | + |

*: protein with the mentioned domain was marked ‘+’ , and protein without the mentioned domain was marked ‘-’.
