# Supplementary material for: Molecular cloning and expression analysis of KIN10 and cold-acclimation related genes in wild banana ‘Huanxi’ (Musa itinerans)
Source: Springerplus. 2015 Dec 30;4:829. doi: 10.1186/s40064-015-1617-z (PMC4695468; doi:10.1186/s40064-015-1617-z)
Supplement: Supplementary file 8 — 10.1186/s40064-015-1617-z Information of primers used for qRT-PCR. [file 40064_2015_1617_MOESM8_ESM.doc]

**Supplemental Table S4** Information of primers used for qRT-PCR

| Gene name | Prime pairs name | Primer sequences(5'-3') | Product length (bp) |
| --- | --- | --- | --- |
| *KIN10* | *KIN10-*F | GAGATTCGAGAACATCCATGG | 206 |
|  | *KIN10-*R | ACTCATAGCACGGAACCTATTG |  |
| *HOS1* | *HOS1-*F | GATCGGCATTGGAAGAGAC | 186 |
|  | *HOS1-*R | GAGAACTTGAGCTATCTTCGG |  |
| *ICE1* | *ICE1-*F | GAACTCCGCAAGCCAATTC | 300 |
|  | *ICE1-*R | CTTCCTCCAATGCAGCCA |  |
| *18S rRNA* | *18S rRNA* -F | CCTGAGAAACGGCTACCACAT | 171 |
|  | *18S rRNA* -R | CACCAGACTTGCCCTCCA |  |
